# Method-Driven Physicochemical Profiling of *Aconitum pendulum* Bush Polysaccharides and Optimization of Extraction Protocols

**DOI:** 10.3390/ph18050760

**Published:** 2025-05-21

**Authors:** Mingkun Meng, Linlin Zhao, Chunqiao Shi, Yuying Song, Qingya Yu, Mengjia Li, Xing Yang, Yue Liu, Tong Xu, Yi Zhang

**Affiliations:** 1 State Key Laboratory of Southwestern Chinese Medicine Resources, School of Pharmacy, Chengdu University of Traditional Chinese Medicine, Chengdu 611137, China; mingkunmeng2022@163.com (M.M.); zhaolinlin0214@stu.cdutcm.edu.cn (L.Z.); songyuying@stu.cdutcm.edu.cn (Y.S.); liuyue2@cdutcm.edu.cn (Y.L.); 2 School of Pharmacy, Chengdu University of Traditional Chinese Medicine, Chengdu 611137, China; 3 Ethnic Medicine Academic Heritage Innovation Research Center, School of Ethnic Medicine, Chengdu University of Traditional Chinese Medicine, Chengdu 611137, China; 4 Chengdu Institute of Chinese Herbal Medicine, Chengdu 610016, China

**Keywords:** *Aconitum pendulum*, polysaccharides, extraction

## Abstract

**Background/Objectives**: This study aimed to characterize the physicochemical properties and antioxidant activities of polysaccharides from Aconitum pendulum Bush processed through different methods (the polysaccharide from A. pendulum (DT), the polysaccharide from A. pendulum processed with zanba (Z-DT), the polysaccharide from A. pendulum processed with highland barley wine (Q-DT), and the polysaccharide from A. pendulum processed with hezi (H-DT)). Additionally, the research focused on optimizing the hot water extraction process for DT using response surface methodology (RSM) to enhance extraction efficiency and establish a scientific basis for pharmaceutical applications. **Methods**: The physicochemical properties and antioxidant activities of the four polysaccharides were systematically evaluated. RSM with a 17-run Box–Behnken design was employed to investigate the extraction process, examining three factors: extraction runs, liquid–solid ratio, and extraction time. **Results**: The physicochemical properties and antioxidant assays demonstrated that the DT exhibited significantly higher properties. The factors influencing the extraction process were ranked as extraction runs > liquid–solid ratio > extraction time. The optimal conditions for DT were a liquid–solid ratio of 25 mL/g, extraction time of 2.5 h, and four extraction runs, yielding a sugar content of 63.4%. Under these conditions, the extraction rate of DT was significantly higher than before optimization. **Conclusions**: The study demonstrated distinct structural features among the four polysaccharides, providing a scientific framework for their potential pharmaceutical applications. What’s more, the optimized hot water extraction protocol for DT was validated for high extraction rate and reproducibility.

## 1. Introduction

*A. pendulum* is a traditional Tibetan medicine, and its active ingredients have important scientific value and application prospects [1]. With demonstrated anti-inflammatory, analgesic, and anti-rheumatic pharmacological activities, *A. pendulum* is effective in the treatment of joint pain [2], bruises, sprains, and muscle strains. *A. pendulum* is rich in alkaloids [3], polysaccharides, and flavonoids [4]. Recently, different chemical constituents of *A. pendulum* have been widely studied, among which the most researched ones are alkaloids, but other types of bioactive substances have not been studied, such as the polysaccharides of *A. pendulum* [5]. It is noteworthy that polysaccharides isolated from the same genus possess significant immunomodulatory [6], anti-rheumatoid Arthritis [7,8], and antitumor activities [9]. Currently, the extraction methods for polysaccharides from *A. pendulum* are still rather crude, with notable deficiencies in extraction efficiency, purity control, and the retention of active components. To fully tap into the application potential of polysaccharides from *A. pendulum*, achieve their efficient utilization, and maximize their resource value, it is urgent to conduct a systematic optimization study on the existing extraction process.

Hot water extraction remains the preferred method for plant polysaccharides due to its operational simplicity and structural preservation capabilities [10]. By comparing the antioxidant and hypoglycemic activities of polysaccharides extracted from Red Pitaya Stem using various methods, it was found that the sample extracted with hot water demonstrated the highest activity [11]. Similarly, *Bletilla striata* polysaccharides obtained through hot water extraction exhibited the highest molecular weight and thermal stability compared to ultrasonic and enzymatic methods [12]. Secondly, in *A. carmichaeli*, hot water extraction produced polysaccharides with greater purity and molecular weight stability than ultrasound-assisted extraction [13]. Natural polysaccharides demonstrate an extremely broad research prospect, and achieving the efficient extraction and rational utilization of polysaccharides will undoubtedly lay a solid foundation for in-depth research on polysaccharide-related fields.

Conducting a systematic study on the extraction methods of polysaccharides from *A. pendulum* not only enables the efficient utilization of resources but also helps establish a comprehensive quality control standard system. In this study, we explored how various types of processing, including processing with zanba (Z-DT) [14], processing with highland barley wine (Q-DT) [15], and processing with hezi (H-DT) [16], influenced the physicochemical properties of polysaccharides derived from *A. pendulum*. The extracts were characterized multidimensionally by a combination of different identification methods and focused on resolving the conformational relationships of polysaccharides obtained from different concoctions. Based on the conformational relationship and polysaccharide rate, the process of raw *A. pendulum* polysaccharide (DT) with the highest rate and relatively optimal physicochemical properties was investigated to obtain the best extraction process. The optimal extraction process was obtained, which provided a scientific basis for the development and utilization of the polysaccharide resources of *A. pendulum*.

## 2. Results and Discussion

### 2.1. Infrared Analysis

**Figure 1:**
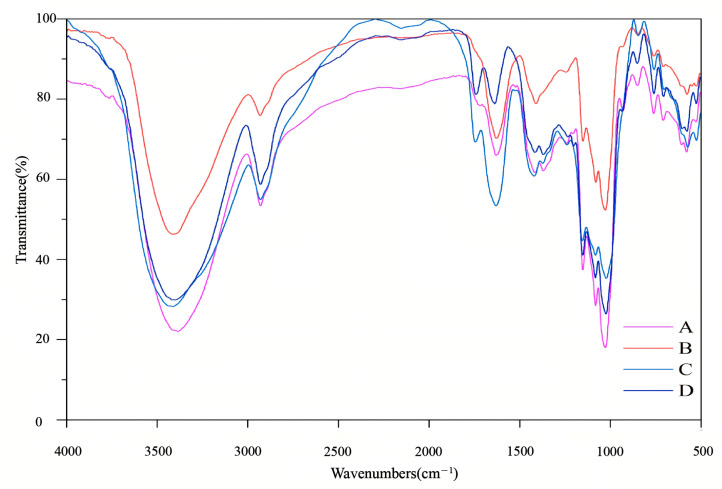
Infrared spectra of *A. pendulum* polysaccharides.

Figure 1 shows the DT, Q-DT, H-DT, and Z-DT infrared absorption spectra. As presented (Figure 1), the FI-IR spectra of polysaccharides from *A. pendulum* from different processes are displayed. The spectral peaks at approximately 3415 cm^−1^ [17] and 2932 cm^−1^ are indicative of the stretching oscillations of the hydroxyl (O-H) and C-H functional groups, respectively [18]. Additionally, the emergence of an asymmetric stretching mode at 1420 cm^−1^ and an absorption feature near 1630 cm^−1^ suggests the incorporation of carbonyl (C=O) and carboxylate (COO^−^) groups into the polysaccharide backbone, a hallmark of acidic polysaccharides [19]. The broad absorption envelope between 1000 and 1200 cm^−1^ arises from coupled vibrations involving C-O/C-C stretching modes and C-OH bending deformations, which are diagnostic of pyranose ring structures, which are the fundamental sugar units in most polysaccharides [20]. These distinctive peaks serve as hallmarks of polysaccharides, implying that Z-DT, H-DT, Q-DT, and DT all exhibit characteristic absorption patterns inherent to polysaccharides. Based on the above analysis, it can be concluded that the differences in the primary structures among different processed products are not significant/are indistinct.

### 2.2. Thermogravimetric Analysis

**Figure 2:**
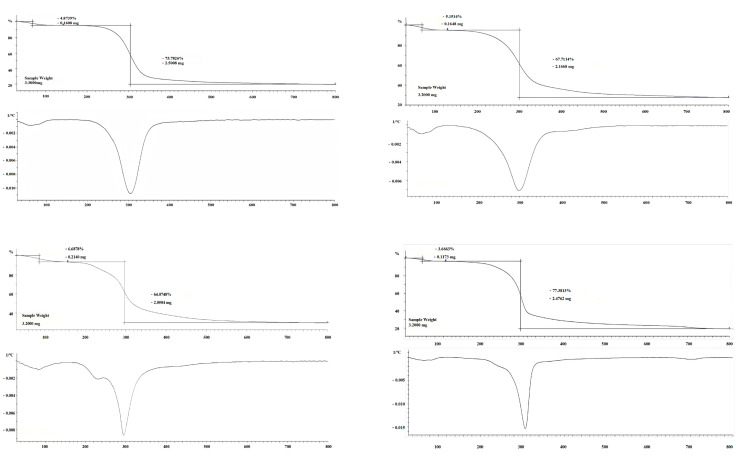
Thermogravimetric analysis of Z-DT (A), Q-DT (B), H-DT (C), and DT (D).

Thermogravimetric analysis (TGA) is used to determine the stability of polysaccharides. The TGA of four polysaccharide samples revealed a slight mass loss within the temperature range of 20–80 °C (Figure 2), which is attributable to the evaporation of physically bound water from the polysaccharide matrices [21]. Remarkably, the H-DT exhibited a significantly elevated temperature threshold during the water evaporation stage, surpassing those observed in the other specimens. (Figure 2C). As the temperature rises, polysaccharides have been found to decompose gradually, mainly at 200–400 °C, and the mass decreases extremely sharply (Figure 2). The mass loss is mainly caused by the thermal decomposition of the polysaccharide backbone at this stage, which consists mainly of glycosidic bond breakage, dehydroxylation, and decarboxylation reactions [22]. The polysaccharide profile stabilizes when the temperature is more than 400 degrees Celsius, at which point the residues are mainly carbonized polysaccharides and impurities. In summary, the thermal stability of the four polysaccharides was good [23].

### 2.3. SEM Analysis

**Figure 3:**
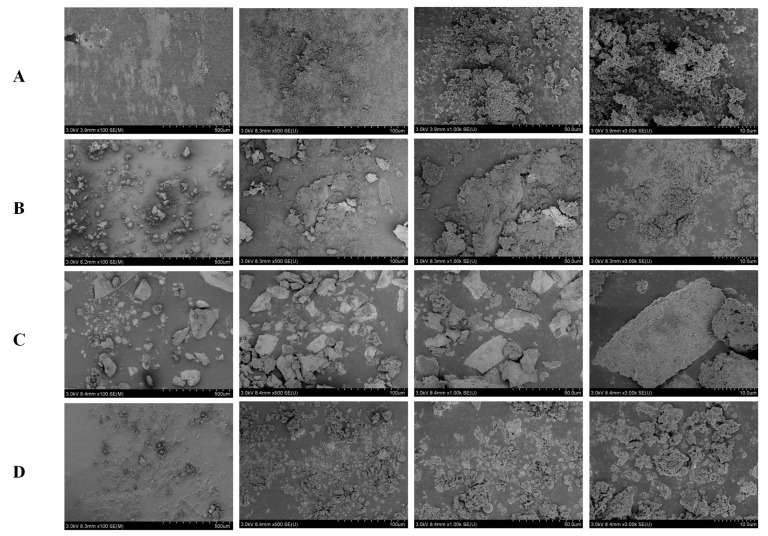
SEM of Z-DT (**A**), Q-DT (**B**), H-DT (**C**), and DT (**D**).

The microstructure of polysaccharides is profoundly affected by the choice of extraction protocols [24]. Scanning electron microscopy (SEM) serves as a robust analytical tool for elucidating the surface features of polymeric materials at the micrometer and sub-micrometer scales. The results indicated that polysaccharides processed via different methods exhibited distinct morphological characteristics (Figure 3). Under low-magnification microscopy, Q-DT and H-DT samples presented as massive aggregates, whereas at high magnification, they displayed a loose, porous structure [25]. In contrast, DT and Z-DT surfaces demonstrated numerous spherical formations with comparatively fewer bulk structures, a characteristic that appears to be associated with their smaller particle dimensions and relative molecular mass differences.

### 2.4. DPPH Radical Scavenging Activity

**Figure 4:**
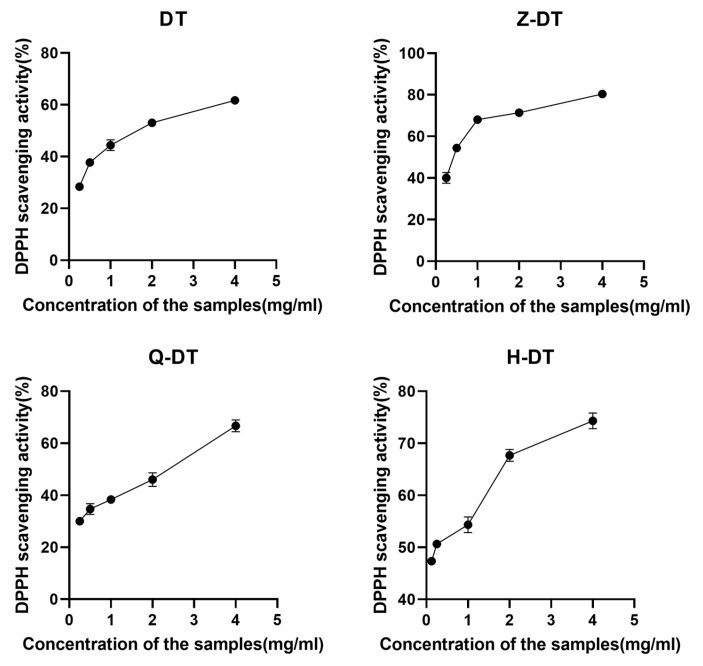
Scavenging activity of polysaccharides from different processing methods on DPPH.

The 2,2-diphenyl-1-picrylhydrazyl (DPPH) radical, an unchanging organic nitroxide derivative, serves as a robust model system for evaluating the free radical-quenching efficacy of antioxidative agents [26]. As depicted in Figure 4, the polysaccharides exhibit a concentration-dependent suppression of DPPH radicals, with significant scavenging activity observed across a dosage spectrum of 0.25–4.0 mg/mL. No statistically significant disparities in DPPH radical-quenching efficacy were observed across the DT, Z-DT, Q-DT, and H-DT treatment groups (*p* > 0.05), implying comparable antioxidative potential against DPPH radicals. However, H-DT demonstrated a markedly lower IC_50_ of 2.0 ± 0.1 mg/mL (*p* < 0.05), signifying its greater capacity to suppress DPPH radicals compared to the remaining samples. In contrast, Q-DT exhibited the highest IC_50_ value (4 mg/mL), indicating the weakest DPPH radical scavenging capacity among the tested samples. The results from Figure 4 indicate that all four types of *A. pendulum* polysaccharides exhibited certain scavenging capacities against DPPH radicals in a concentration-dependent manner. However, their scavenging abilities were weaker than that of vitamin C (VC) (Figure S1). Additionally, the study found that DT had the highest extraction rate (Table S1).

### 2.5. Effect of Single Factor on the Extraction Rate of DT

**Figure 5:**
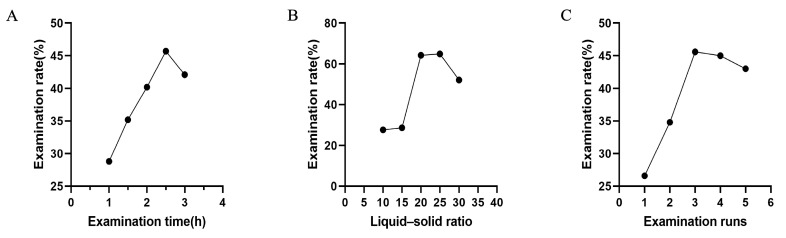
(**A**) The impact of extraction time on the rate of DT extraction. (**B**) The impact of extraction runs on the rate of DT extraction. (**C**) The impact of the liquid–solid ratio on the rate of DT extraction.

One of the critical parameters governing polysaccharide rate is the liquid–solid ratio. As illustrated in Figure 5B, a positive correlation was observed between the DT extraction efficiency and liquid–solid ratios ranging from 10 to 20 mL/g of distilled water. Increasing the liquid–solid ratio within this interval enhances the concentration gradient across plant cell membranes, thereby accelerating the diffusion of intracellular solutes and promoting polysaccharide dissolution. The optimum liquid–solid ratio was resolved to be 20 mL/g. Beyond this threshold, although the extraction rate increased, the extraction efficiency significantly declined compared to the range of 10–20 mL/g. This phenomenon is likely attributed to the excessive solvent volume, which impedes temperature elevation during the extraction process and reduces the dissolution rate of intracellular polysaccharides. The impact of extraction time and extraction runs on the extraction rate of DT polysaccharides can be discerned from Figure 5A,C. Within a defined temporal range, the extraction rate of DT initially exhibited a marked increase, followed by a subsequent decline. This phenomenon may result from a combination of factors, including the incomplete disruption of plant cell walls due to insufficient extraction time at the early stage, the degradation of DT during prolonged extraction, and the re-adsorption of DT onto plant residues or impurities. With an increase in the number of extraction runs, the polysaccharide rate initially increased rapidly, reaching a maximum after three extraction runs. While the rate continued to rise at the fourth run, the rate of increase slowed significantly, likely due to the co-elution of impurities that hindered polysaccharide release. Based on these observations, three extraction runs were selected as the optimal condition. In summary, the best conditions derived from the single-factor experiments were identified as a liquid-to-solid ratio of 20 mL/g, an extraction time of 2.5 h, and three consecutive extraction runs.

### 2.6. Determination of Response Surface Optimization Hot Water Extraction and Results

In order to obtain the optimal extraction process, this study used Design-Expert 13 statistical analysis software to establish a three-factor, three-level response surface test based on single-factor experimental results (Table 1).

### 2.7. Model Building and ANOVA

Software fitting and analysis produced the quadratic multinomial regression equation of DT extraction rate(X) time(A), runs(B), and versus liquid–solid(C), and the following equations were obtained: X = 56.88 + 2.5*A + 5.2*B + 2.85*C + 2.1*A*B + 1.15*B*C + 2.2*A*C − 3.21*A^2^ − 3.82*B^2^ − 2.66*C^2^. The experimental results are offered in Table 2. The model confirmed statistical significance with a value of *p* < 0.05, while the lack-of-fit term exhibited a *p* = 0.1391 (>0.05), indicating the reliability of the experimental model. The model demonstrated excellent predictive performance, with predicted values closely matching experimental observations. The R^2^ was 0.9839, indicating that 98.39% of the variability in the extraction rate was explained by the model. This high R^2^ value validates the model’s suitability for predictive analysis within the experimental range. In the regression model, none of the linear terms touched statistical significance (*p* > 0.05). Among the interaction terms, all except the BC interaction were statistically significant (*p* < 0.05). Additionally, both quadratic terms for A^2^, B^2^, C^2^ exhibited significant effects (*p* < 0.05). The F-values for each factor (Table 2) revealed their relative significance on the extraction rate, ranked as B > C > A (i.e., extraction runs > liquid-to-solid ratio > extraction time). This ordering indicates that the extraction runs (B) had the strongest influence on the extraction rate, followed by the liquid-to-solid ratio (C), and then extraction time (A).

### 2.8. Response Surface and Contour Analysis

**Figure 6:**
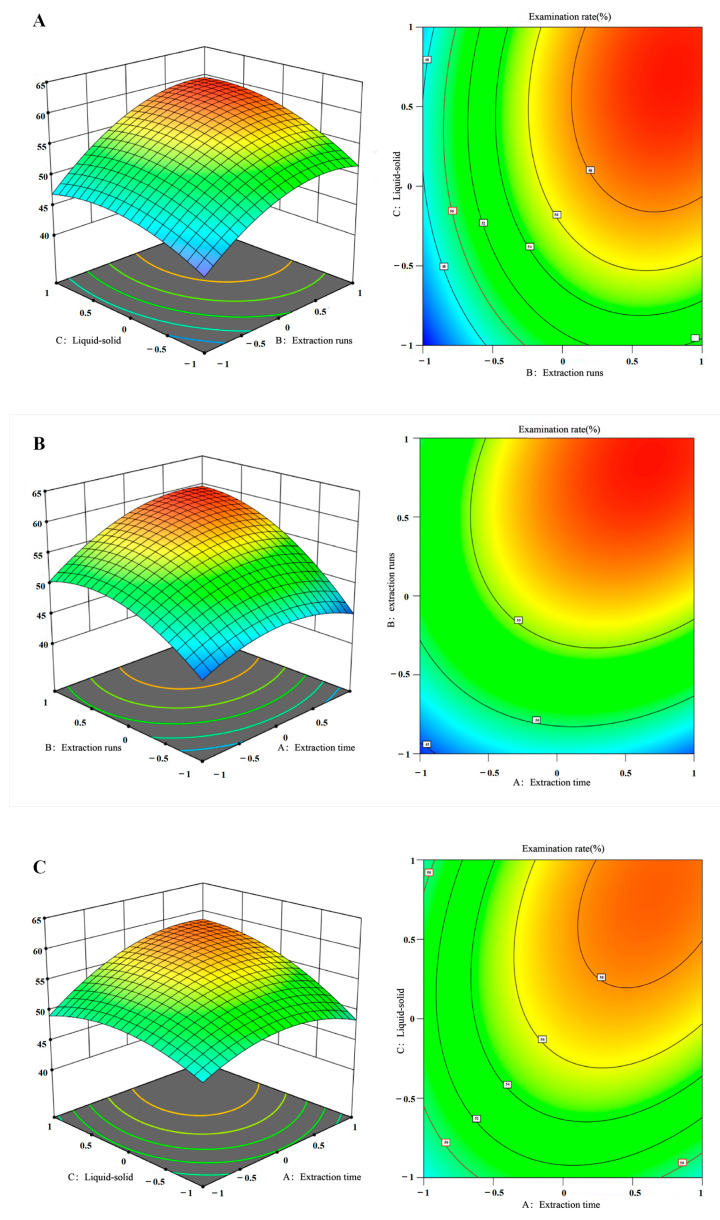
Response surface and contour plots in three dimensions illustrate the interactions between (**A**) the liquid–solid ratio and extraction runs; (**B**) the extraction runs and extraction time; and (**C**) the liquid–solid ratio and extraction time.

The two-by-two interaction of A (extraction time), B (extraction runs), and C (liquid–solid ratio) on the extraction rate can be reflected in Figure 6. The peak prediction value of the three-dimensional response surface is localized within the least elliptically distorted region of the contour plots. Furthermore, the ellipticity of the contour lines intensifies with the growing significance of the interactions among independent variables, reflecting a stronger nonlinear coupling effect. As shown in Figure 6, a three-factor, three-level Box–Behnken design was employed to optimize polysaccharide extraction from *A. pendulum*. The response surface plot reveals a steeper gradient near the optimal region and elliptical contour lines, suggesting significant interactions between parameters. Statistical analysis (ANOVA, *p* < 0.05) confirms that the number of extraction runs (F = 216.32, *p* < 0.001) has the most pronounced effect on the extraction rate, followed by solvent type, and then extraction time.

### 2.9. Optimal Process Validation

The response surface methodology (RSM) predicted the following optimal extraction conditions for polysaccharides from *A. pendulum*: four extraction runs, an extraction time of 3 h, and a liquid–solid ratio of 25 mL/g. The predicted extraction rate of these polysaccharides was 63.19%. To validate the optimized extraction process, 100 g of *A. pendulum* powder was accurately weighed into a round-bottomed flask and subjected to extraction under the best process conditions. The actual extraction rates of the polysaccharides were 62.76%, 63.47%, and 63.98%, with a relative standard deviation (RSD) value of 0.96%. This result demonstrated the stability and reliability of the optimized extraction conditions derived from the response surface experiment.

## 3. Materials and Methods

### 3.1. Materials

The roots of *A. pendulum* were collected in September from Huzhu Tu Autonomous County, Haidong City, Qinghai Province, and then dried at a temperature of 40 °C. They were authenticated by Professor Yi Zhang from the School of Ethnic Medicine, Chengdu University of Traditional Chinese Medicine (Chengdu, China). H_2_SO_4_, anhydrous ethanol, and KBr were purchased from Chengdu Colony Chemicals Co., Ltd. (Chengdu, China).

### 3.2. Pretreatment of the Sample

The processing of *A. pendulum* with zanba [14], the processing of *A. pendulum* with highland barley wine [15], and the processing of *A. pendulum* with hezi [16] were carried out according to the methods previously investigated by our research group. Dried *A. pendulum*, the processing of *A. pendulum* with zanba, the processing of *A. pendulum* with highland barley wine, and the processing of *A. pendulum* with hezi were first powdered using a mechanical grinder, sieved (100 mesh), and subsequently subjected to the following sequential solvent extraction processes: degreased with petroleum ether and decolorized with ethanol to obtain the refined plant material. The resulting residues were dried to a constant weight.

### 3.3. Hot Water Extraction

The powder derived from Section 3.2 was put in water at a liquid–solid ratio of 15 mL/g and subjected to hot-water extraction (100 °C) in a thermostatic water bath for 2 h. The polysaccharide solution was obtained through filtration and concentration. Anhydrous ethanol was added to adjust the final ethanol concentration to 80% (*v*/*v*), followed by overnight at 4 °C. The precipitate was collected via centrifugation, subjected to protein removal using the Sevage method, and subsequently dialyzed against deionized water to rate the purified polysaccharide solution. The polysaccharides obtained via freeze-drying were categorized into the following four types: the polysaccharide derived from *A. pendulum* (DT), the polysaccharide from *A. pendulum* processed with zanba (Z-DT), the polysaccharide from *A. pendulum* processed with highland barley wine (Q-DT), and the polysaccharide from *A. pendulum* processed with hezi (H-DT).

### 3.4. FT-IR Characterization

Fourier transform infrared (FTIR) spectroscopy (Nicolet 6700, Madison, WI, USA) has been extensively employed for the structural elucidation of polysaccharides. The polysaccharide was mixed with potassium bromide (KBr), pressed into a pellet, and analyzed by FTIR spectroscopy.

### 3.5. In Vitro Antioxidant Activity Test

Human diseases are predominantly linked to the excessive accumulation of free radicals, which induce oxidative stress by disrupting cellular redox homeostasis [26]. Research has demonstrated that plant-derived polysaccharides exhibit robust antioxidant activities, which are primarily attributed to their capacity to scavenge free radicals [27]. To assess the antioxidant potential of four polysaccharides (Z-DT, Q-DT, H-DT, and DT), this study employed the 2,2-diphenyl-1-picrylhydrazyl (DPPH) radical-scavenging assay.

### 3.6. Thermal Analysis

Thermogravimetric (TG) analysis (Mettler Toledo TGA2+, Zurich, Switzerland) was performed to characterize the thermal decomposition behavior of polysaccharide samples using a high-resolution synchronous thermal analyzer. Samples were placed in pre-weighed alumina crucibles, with an empty aluminum pan serving as the inert reference. The TG curves were recorded under a N_2_ atmosphere while heating from 30 °C to 800 °C at a constant rate of 10 °C/min.

### 3.7. Scanning Electron Microscopy (SEM)

The microstructural morphology of the sample was characterized using an SEM (Hitachi 9278-02, Tokyo, Japan). The specimens were securely affixed to the sample stage, followed by gold sputter-coating to enhance surface conductivity, and were subsequently examined under varying magnifications using scanning electron microscopy.

### 3.8. Determination of Sugar Content

**Figure 7:**
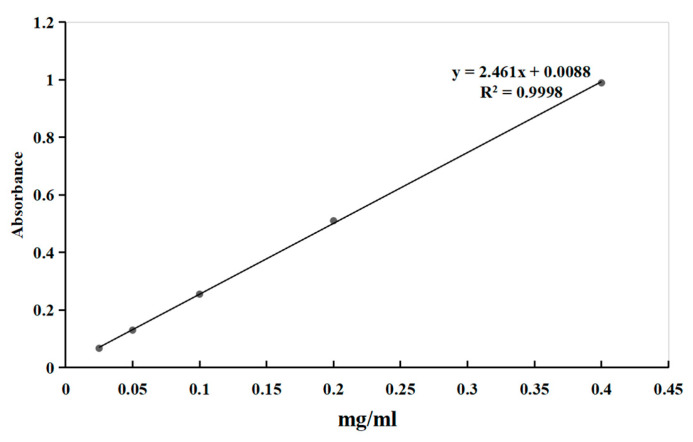
The standard curve of absorbance versus total polysaccharides (mg/mL).

The sugar content was determined using the sulfuric acid–phenol method. An amount of 10 mg of glucose reference standard substance was weighed and subsequently prepared into a 1 mg/mL standard solution. Amounts of 0.25, 0.5, 1, 2, and 4 of the 1 mg/mL anhydrous glucose solution were accurately added into a 10 mL volumetric flask, and deionized water was added to the scale. Amounts of 1 mL of glucose (0.025, 0.05, 0.1, 0.2, 0.4 mg/mL) were added into tubes. The standard curve was constructed using the phenol–sulfuric acid method. Briefly, 0.5 mL of standard solution was mixed with 0.5 mL of 5% (*w*/*v*) phenol solution and 2.5 mL of concentrated sulfuric acid (95–98%, *v*/*v*). The mixture was vortexed thoroughly, cooled to room temperature, and the absorbance was measured at 490 nm. The linear regression equation relating absorbance to total sugar content was derived as y = 2.4639x + 0.0051 (R^2^ = 0.9998) (Figure 7).

The polysaccharide content in samples was determined using the same analytical protocol as the standard curve construction. Briefly, the absorbance of each sample was measured at 490 nm against a reagent blank. The measured absorbance values were then substituted into the previously established linear regression equation (y = 2.4639x + 0.0051, R^2^ = 0.9998) to calculate the corresponding total sugar concentration.

### 3.9. DPPH Scavenging Activity Assay

The appropriate amount of polysaccharide powder was weighed and dissolved with water to obtain 0.4 mg/mL of test material, and it was diluted sequentially backward. An amount of 4.0 mg of DPPH solution was weighed, and 50 mL of anhydrous ethanol was added to dissolve. A solution with a concentration of 0.00625 mg/mL was prepared by dissolving vitamin C (VC) in absolute ethanol, and subsequent serial dilutions were then carried out. The experimental group was divided into negative control group A (150 μL of anhydrous ethanol and 50 μL of DPPH solution), sample group B (150 μL of sample or positive control VC and 50 μL of DPPH solution), and sample control group C (150 μL of sample and 50 μL of anhydrous ethanol) and was added into a 96-well plate sequentially. The absorbance was measured at 517 nm with an enzyme meter after 30 min of full reaction protected from light. After three runs in parallel, the DPPH radical scavenging rate was calculated (scavenging rate = [A − (B − C)]/A).

### 3.10. Single Factor Experiment

#### 3.10.1. Examination of Liquid–Solid Ratio

In this study, we conducted an examination of the liquid–solid ratio to determine its effect on the extraction efficiency. The following five different liquid–solid ratios were used: 10, 15, 20, 25, and 30 g/mL. The results are expected to provide insights into the optimal ratio for achieving the highest extraction rate.

#### 3.10.2. Examination of Extraction Time

In this study, we conducted an extraction time examination to determine its effect on the extraction efficiency. The following five different time were used: 1 h, 1.5 h, 2 h, 2.5 h, and 3 h. The results are expected to provide insights into the optimal ratio for achieving the highest extraction rate.

#### 3.10.3. Examination of Extraction Runs

In this study, we conducted an examination of the effect of extraction runs on the extraction efficiency from *A. pendulum* power with hot water as the solvent. The following five different extraction runs were used: 1, 2, 3, 4, and 5. The results are expected to provide insights into the optimal ratio for achieving the highest extraction rate.

### 3.11. Optimization of Experimental Conditions

To systematically investigate the optimal conditions conducive to the hot water extraction process of DT, the following three pivotal parameters were discerned through the application of Response Surface Methodology (RSM): the liquid–solid ratio (A), extraction runs (B), and extraction time (C). As delineated in Table 3, a three-factor, three-level experimental design was constructed utilizing the Box–Behnken central composite design framework, which serves as a well-established and statistically robust approach for such multi-parameter optimization studies.

## 4. Conclusions

In this study, the hot water extraction method was used to extract *A. pendulum* polysaccharides processed from Zanba, Qinke wine, and Hezi soup, and the rate, structural characterization, stability, and antioxidant activity of the polysaccharides were investigated. The results showed that DT had better thermal stability than the other three polysaccharides. The extraction process of DT was optimized to obtain the optimal extraction process. The foundation was laid for the future application of *A. pendulum* in the pharmaceutical field.

## Figures and Tables

**Table 1:** Response surface optimization design and results.

Run	A: Extraction Time (h)	B: Extraction Runs	C: Liquid–Solid Ratio (mL/g)	Extraction Rate (%)
1	−1	−1	0	43.5
2	1	−1	0	44.3
3	−1	1	0	51.2
4	1	1	0	60.4
5	−1	0	−1	48.3
6	1	0	−1	48.9
7	−1	0	1	48.7
8	1	0	1	58.1
9	0	−1	−1	43.8
10	0	1	−1	50.4
11	0	−1	1	48.1
12	0	1	1	59.3
13	0	0	0	57.3
14	0	0	0	57.1
15	0	0	0	56.4
16	0	0	0	57.8
17	0	0	0	55.8

**Table 2:** Variance and significance analysis of the response surface quadratic regression equation.

Source of Variation	Sum of Squares	Degree of Freedom	Mean Square	F Value	*p* Value	Significance
Model	523.68	9	58.19	47.43	<0.0001	**
A	50	1	50	40.75	0.0004	**
B	216.32	1	216.32	176.32	<0.0001	**
C	64.98	1	64.98	52.96	0.0002	**
AB	17.64	1	17.64	14.38	0.0068	**
AC	19.36	1	19.36	15.78	0.0054	**
BC	5.29	1	5.29	4.31	0.0765	*
A^2^	43.52	1	43.52	35.47	0.0006	**
B^2^	61.28	1	61.28	49.95	0.0002	**
C^2^	29.9	1	29.9	24.37	0.0017	**
Residual	8.59	7	1.23			
Lack of Fit	6.12	3	2.04	3.31	0.1391	
Pure Error	2.47	4	0.617			
Total	532.26	16				
R^2^	0.9839					
R_adj_^2^	0.9631					

Note: * indicates a statistically significant difference (*p* < 0.05); ** indicates a highly statistically significant difference (*p* < 0.01).

**Table 3:** Experimental factor levels.

Factor	Levels		
	−1	0	1
A: Runs	2	3	4
B: Time/h	2	2.5	3
C: Liquid–solid ratio/mL/g	15	20	25

## Data Availability

Data will be made available upon request.

## Supplementary Materials

The following supporting information can be downloaded at: https://www.mdpi.com/article/10.3390/ph18050760/s1, Figure S1. Scavenging activity of VC on DPPH. Table S1. Extraction rate of four polysaccharides.
