# Supplementary material for: Method-Driven Physicochemical Profiling of Aconitum pendulum Bush Polysaccharides and Optimization of Extraction Protocols
Source: Pharmaceuticals (Basel). 2025 May 21;18(5):760. doi: 10.3390/ph18050760 (PMC12115121; doi:10.3390/ph18050760)
Supplement: Supplementary file 1 [file pharmaceuticals-18-00760-s001.zip › pharmaceuticals-3619236-supplementary.pdf]

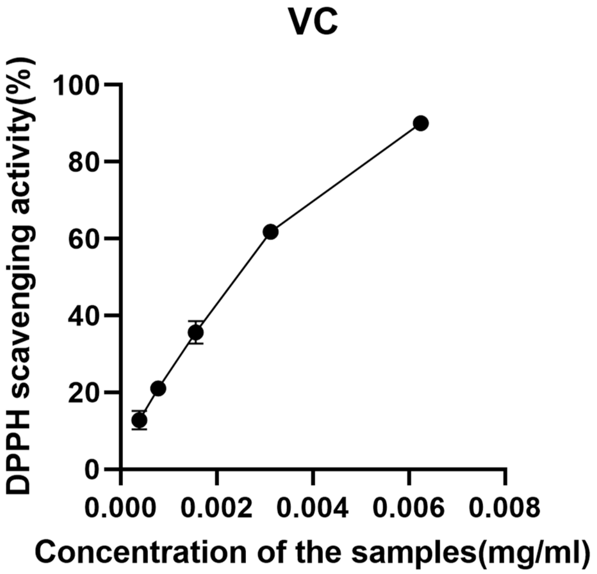

Figure S1. Scavenging activity of VC on DPPH.

Table S1 Extraction rate of four polysaccharides

	DT	Z-DT	Q-DT	H-DT
	34%	28.1%	20.5%	24.3%
	35.7%	29.7%	20.4%	25.5%
	34.3%	27.8%	18.9%	23.8%
$\bar{x}$±SD	0.3467±0.0091	0.2853±0.0102	0.1993±0.009	0.2453±0.0087
